# Factors influencing multiple non-utilised healthcare appointments from patients’ and healthcare providers’ perspectives: a qualitative systematic review of the global literature

**DOI:** 10.3399/BJGPO.2024.0075

**Published:** 2024-12-11

**Authors:** Asrar Aldadi, Kathryn A Robb, Andrea Williamson

**Affiliations:** 1 Taif University, PhD Student at School of Health and Wellbeing, University of Glasgow, Glasgow, UK; 2 School of Health and Wellbeing, University of Glasgow, Glasgow, UK; 3 General Practice and Primary Care, School of Health and Wellbeing, University of Glasgow, Glasgow, UK

**Keywords:** multiple no-show patients, qualitative research, systematic review, lost to follow-up

## Abstract

**Background:**

The term 'non-utilised appointments' emerged in 2019 but lacks a clear definition. We focus on multiple non-utilised appointments owing to recent advances in understanding 'missingness' in UK health care. Studies on missed appointments show conflicting results regarding interventions such as text messaging owing to oversight of occasional versus repeated missed appointments. Understanding patient and healthcare-related factors in multiple non-utilised appointments is crucial for improving interventions and patient engagement.

**Aim:**

To identify factors influencing multiple non-utilised appointments from patients' and healthcare providers' perspectives.

**Design & setting:**

A systematic review of qualitative research identifying factors that influence multiple non-utilised appointments across diverse global healthcare settings.

**Method:**

The review employed a qualitative systematic approach, encompassing diverse papers from multiple databases, irrespective of patient or healthcare provider age, location, or setting. Data analysis followed Thomas and Harden’s thematic synthesis method. Themes are presented in alignment with both the health service and patient perspective aspects of the Levesque access model.

**Results:**

Ten thousand and eighty-six records were retrieved. Five studies met the inclusion criteria and were analysed. Six key themes influenced appointment utilisation. Healthcare system determinants highlighted provider–patient relationship and professionalism, and healthcare organisation factors role in appointment utilisation. Patient experience and decision making explored personal factors. Additionally, communication, support, and engagement delved into challenges with communication and language, family and social support, and socio-familial barriers to appointment utilisation. Health and wellbeing factors encompassed medical conditions, mental and emotional factors, and psychosocial determinants affecting appointment utilisation. Moreover, financial constraints and socioeconomic factors were identified as significant contributors. Lastly, healthcare access and barriers addressed transportation challenges, accessibility issues, and geographical barriers impacting healthcare access.

**Conclusion:**

The analysis reveals complex factors influencing multiple non-utilised appointments. Strong provider–patient relationships improve care accessibility. Flexible scheduling and patient-centred approaches are pivotal, alongside addressing workplace discrimination. Tailored healthcare services and overcoming geographical barriers are essential. Ensuring safety, accessibility, and communication, while supporting vulnerable groups and mental health needs, are necessary. Equitable access to services and alternative transportation solutions are essential for comprehensive healthcare delivery.

## How this fits in

Previously, various terms such as 'no-show' and 'missed appointments' were used to describe appointments that did not occur as planned. However, the term 'non-utilised appointments' is adopted to comprehensively address this issue using less stigmatising language, with a specific emphasis on 'multiple' non-utilised appointments. The concept of missingness, owing to recent advances in understanding, is important in making this distinction. This systematic review of the global qualitative evidence provides a more nuanced understanding of the underlying and complex reasons for missingness at the patient level. The research underscores the importance of healthcare system factors, patient experiences, communication, health and wellbeing, financial and socioeconomic considerations, as well as healthcare access in influencing the occurrence of multiple non-utilised appointments. These insights offer valuable guidance for healthcare providers aiming to enhance appointment utilisation and patient engagement.

## Introduction

The concept of 'missingness' in health care has recently been conceived. It is defined as the ‘*repeated tendency not to take up offers of care that have a negative impact on the person and their life chances*’,^1^ and which seek to advance how we think about health services access and use. This underscores the importance of understanding the reasons why scheduled appointments were not utilised. Various terms such as 'no-show', 'missed appointments', 'patient absenteeism', 'non-adherence', 'lost-to-follow-up', and 'non-compliance' have been used over time^2–10^ to describe the issue, but some may unfairly blame patients or overlook structural factors.

The term 'non-utilised appointments' was introduced in 2019 to encompass appointments that do not occur as planned, whether owing to patient or provider actions. This covers no-show events and appointments that went unused.^11^ The literature lacks consensus on a precise definition of 'non-utilised appointments'. However, we adopt 'non-utilised appointments' as a comprehensive less stigmatising term,^12^ shifting the emphasis away from labelling and blaming the patient, aiming to acknowledge and hence address various factors beyond the patient’s control.

Building on recent advances about 'missingness' in health care we focus on 'multiple' non-utilised appointments, defined as appointments made but not occurring twice or more times as scheduled, attributable to the individual patient. This shift from 'missingness' to 'non-utilised appointments' offers a more focused framework. It provides clarity and establishes a measurable metric for addressing appointment non-utilisation. Moreover, this terminology recognises the shared responsibility between patients and healthcare providers. By moving beyond blame, it aims to reduce stigma and foster empathy towards the diverse factors contributing to appointment non-utilisation. In this paper, we used a range of terminology to refer to non-utilised appointments, reflecting the various terms used by authors in the original literature.

From the quantitative evidence, multiple missed appointments are associated with poorer health and social disadvantage.^13^ Missing multiple medical appointments, including preventive care appointments, may be indicative of low engagement.^14^ A higher rate of missed appointments is associated with poorer health outcomes,^15–17^ and a higher rate of hospitalisation compared with appointments attended.^17^ There was an association between more missed appointments and increased visits to the emergency department (ED) in one study;^15^ however, no correlation was found between missed multiple GP appointments and ED attendance in another large population study.^18^ Furthermore, patients who consistently miss appointments raise concerns among clinicians, as they are less likely to adhere to age-appropriate preventive health services.^15^ For instance, 46% of patients who missed two or more appointments in a year had one or more chronic conditions, and 17% had four or more. Patients with multiple long-term conditions are at a heightened risk of missing multiple appointments, particularly those with both physical and mental health diagnoses. Specifically, those with long-term physical health conditions who miss GP appointments twice or more yearly see a threefold increase in all-cause mortality; patients with mental health conditions who miss over two GP appointments face an eightfold increase in risk of death from any cause. Notably, factors beyond natural occurrences, such as suicide, contributed to premature mortality in this patient population.^19^

Demographic factors implicated in multiple non-utilised appointments are complex, with different factors emerging in different samples. Evidence on which age group tends to miss multiple appointments is mixed.^14,16,20^ Sex differences exist, women visit the GP more often and miss multiple appointments, while men miss a higher proportion overall.^14^ Studies on diabetes show that women with type 2 diabetes often miss multiple appointments.^20^ Social determinants of health consistently play a role, including frequent changes in residence,^20^ residence in disadvantaged areas,^16,20^ lower socioeconomic status,^14,21^ and individuals without a partner.^22^ One study found individuals with higher socioeconomic status may miss appointments more owing to their use of private health care alongside public sector appointments.^22^ Furthermore, factors such as fracture history,^22^ prescribed medication, antidepressant use, and poor adherence to medication refill contribute to multiple missed appointments.^16^ Additionally, a higher number of scheduled GP and hospital appointments increased the likelihood of multiple non-attendance.^22^ However, the Kaiser Permanente Northern California Diabetes Registry found that fewer scheduled appointments were linked to more missed appointments, contradicting other studies.^16^

Health service factors also significantly contribute to patients missing multiple appointments. In the UK, general practices with many appointments scheduled 2–3 days in advance often see high rates of multiple non-attendance. Urban practice settings have higher missed appointment rates compared with rural ones.^14^ Repeated missed appointments have been found to be correlated with higher out-of-pocket patient payments for outpatient appointments,^16^ use of a medical interpreter,^15^ and Medicaid insurance.^15,21^ Patients with repeated missed appointments were also found to have fewer bus transfers and shorter bus travel times. However, there were no significant differences in terms of distance to the appointment location and travel time by car.^21^

This qualitative systematic review explores patients' and providers' perspectives on factors contributing to multiple non-utilised appointments globally. By incorporating both perspectives, we aim to comprehensively grasp the factors influencing multiple non-utilised appointments; defined as those unused twice or more. This approach enables us to uncover unique insights into how patient-level challenges differ from rescheduled, delayed, or single missed appointments. We use Levesque’s framework, which views healthcare access as a complex interplay involving services, providers, systems, organisations, and patient environments,^23^ to explore these perspectives thoroughly.

### Levesque’s conceptual framework

Levesque’s conceptual framework, established in 2013 after extensive literature review, is widely used in healthcare access research.^24^ It considers both healthcare system and patients' perspectives, defining access as the capability to identify needs, seek services, and receive appropriate care. It includes supply (healthcare system) and demand (patients’ experiences and/or perspectives) aspects, facilitating action throughout the care process. The framework outlines five dimensions of accessibility: approachability, acceptability, availability and accommodation, affordability, and appropriateness. These dimensions interact with five corresponding abilities of populations: ability to perceive, seek, reach, pay, and engage.^25^ By utilising the Levesque conceptual framework, we aim to untangle the intricate mix of factors contributing to multiple non-utilised appointments.

## Method

The Preferred Reporting Items for Systematic Reviews and Meta-Analyses (PRISMA) statement was utilised in this review.^26^ In addition, the review was reported in accordance with the Enhancing Transparency in Reporting the Synthesis of Qualitative Research (ENTREQ) statement.^27^

### Eligibility criteria

The inclusion and exclusion criteria can be seen in Table 1.

**Table 1.** Criteria (inclusion, exclusion) of research questions

Category	Inclusion criteria	Exclusion criteria
Population	All types of patients and healthcare provider of any age	–
Setting	Studies from any country and any setting	–
Study design	Qualitative	All other study designs including those where there was a qualitative component to mixed methods
Outcomes	Studies related to multiple non-utilised appointments; for example, repeated missed appointments, repeated non-attendance, repeated no-show, repeated non-compliance, multiple broken appointment, repeated non-adherence, repeated non-utilised appointment, multiple lost to follow-up related to appointment, and repeated absenteeism studies	Studies not related to the 'non-utilised appointments' Cancelled operation studies, to concentrate specifically on booked appointments. Cancelled operations involve different procedures and are not directly related to booked appointments Regular follow-up studies, which measure follow-up appointments without any missed visits, were excluded. This decision was made to ensure that our focus remains on non-utilised appointments, avoiding potential overlaps with studies that do not involve missed appointments Medication adherence was excluded as it represents a distinct topic that does not necessarily require a booked appointment and is not directly linked to the utilisation of such appointments
Language	Studies published in English	Other languages
Availability of text	Full-text studies	Studies where full text is unavailable despite contacting the authors
Publication date	Open to any publication date	–
Publication type	Peer-reviewed articles	Grey literature was excluded owing to time constraints. These types of literature may lack the same level of quality and robustness as materials that undergo peer review

### Information sources

Five electronic databases (Embase, PubMed, CINAHL**,** Ovid MEDLINE, and PsycInfo) were searched.

### Search strategy

We used Boolean operators and syntax elements (for example, asterisks, parentheses, OR, and AND) in our search (for example, 'Non-attend* OR Non-adhere* OR No-show OR Non-compliance OR Broken OR Missed OR lost to follow-up OR Absenteeism OR Non-utilised OR failure to attend OR not brought OR did not attend AND [Qualitative] AND Appointments'). Initially, our searches yielded diverse literature, documented in the log. Adding 'qualitative' and related terms refined the results, significantly reducing returned records. We used free text and manual searches, resulting in five studies.

### Selection process

We eliminated duplicates, assessed titles and abstracts, included relevant papers meeting our criteria, and conducted full-text reviews. To ensure accuracy, AW and KAR cross-checked 10% of titles and abstracts.

### Data collection and quality assessment

AA extracted details from studies using Excel. AA, AW, and KAR independently assessed risk of bias using Critical Appraisal Skills Programme (CASP) checklist, discussing discrepancies for a final score.^28^

### Synthesis methods

Thematic synthesis was conducted by AA using Thomas and Harden’s thematic analysis, including coding, developing descriptive themes, and generating analytical themes.^29^ NVivo software (version 12) was used for coding and analysing. AA coded the text line by line, distinguishing between data reported by the original authors (descriptive theme) and additional analysis. These additional analyses included themes newly identified from the participants' quotations in the papers. Descriptive themes were identified by recognising commonalities among all codes. Additional analyses included themes newly identified from the participants' quotations in the papers AA identified. In the analytical phase, relationships between descriptive themes were identified to create analytical themes, providing insight into factors contributing to multiple missed appointments. These themes align with both the supply (healthcare system) and demand (patients' experience and/or perspectives) aspects of the Levesque access model. All themes were discussed during supervision meetings.

## Results

### Search results

**Figure 1.**
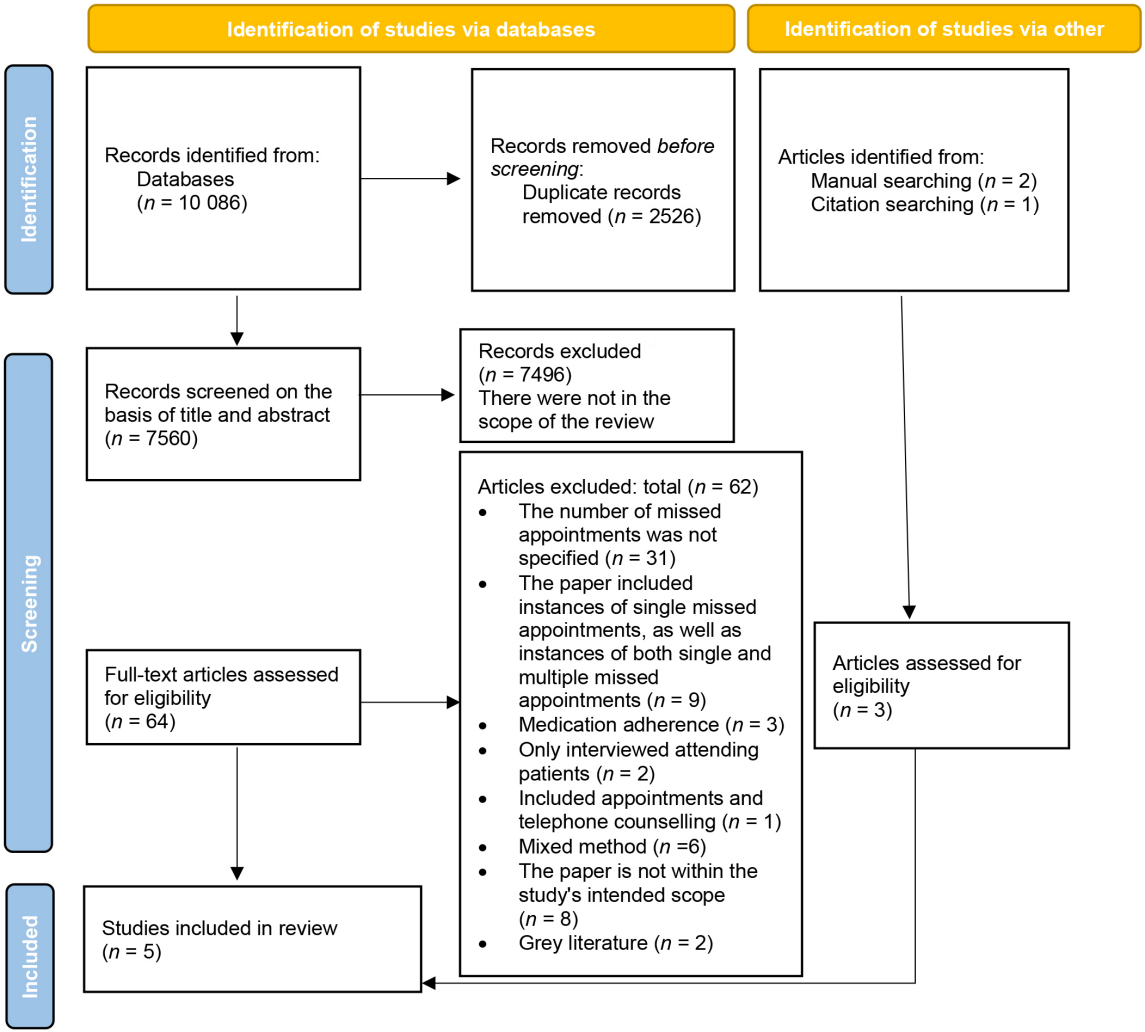
Study selection flow diagram

A total of 10 086 articles were identified from databases, with 7560 unique articles remaining after removing duplicates. After title and abstract screening, 64 full-text articles were assessed, resulting in the inclusion of five studies that met the inclusion criteria. The PRISMA flow diagram shows an overview of this paper-selection process (Figure 1).

### Study characteristics

The studies included a range of healthcare settings and countries: an HIV clinic in South Africa,^30^ family medicine residency clinics in the US,^31^ a paediatric rehabilitation hospital in Canada,^32^ a migrant health clinic in Denmark,^33^ and diabetic retinopathy screening in the UK.^34^ In total, 151 patient and professional participants were involved, and diverse data collection and analysis methods were used (Supplementary Table S1).

### Risk of bias in studies

The potential bias in each of the included studies was evaluated using the CASP checklist (Table 2). Following this, one study was categorised as high quality,^33^ two studies were rated as moderate quality,^32,34^ and two were determined to be of low quality.^30,31^

**Table 2.** Summary of risk of bias using CASP

Study citation	Scoring system	Limitations
Lowane and Lebese^30^	Low quality	Lack of a theoretical framework Insufficient description of the recruitment strategy, data collection, and data analysis Ethical approval was obtained with limited detailed information provided Lack of author reflexivity and positionality
Ofei-Dodoo *et a*l^31^	Low quality	Lack of a theoretical framework Insufficient description of the recruitment strategy, data collection, and data analysis Ethical approval was obtained with limited detailed information provided Lack of author reflexivity and positionality
Ballantyne *et al* ^32^	Moderate quality	Lack of a theoretical framework Selection bias Lack of author reflexivity and positionality Ethical approval was obtained with limited detailed information provided
Hipwell *et al* ^34^	Moderate quality	Lack of author reflexivity and positionality Inadequate information regarding the interpretation and analysis of the data
Abdulkadir *et al* ^33^	High quality	Lack of author reflexivity and positionality

CASP = Critical Appraisal Skills Programme.

### Review findings

Four studies^30–33^ encompassed all analytical themes and aligned with both the supply (healthcare system) and demand (patients' experiences and/or perspectives) aspects of Levesque’s access model (Table 3).

**Table 3.** Factors identified as influencing multiple non-utilised appointments

Analytical themes	Descriptive themes	Levesque’s access model	
		Supply-side (healthcare system)	Demand-side (patients' experiences and/or perspectives)
Healthcare system determinants	Provider–patient relationship and professionalism	^30^^,^^32^^,^^34^	^30^
	Healthcare organisation factors	^30^^,^^31^^,^^32^^,^^33^^,^^34^	
	Safety determinants of appointment utilisation		^30^
Patient experience and decision making	Personal factors		^30^^,^^31^^,^^32^^,^^33^^,^^34^
Communication, support, and engagement	Challenges with communication and language		^33^
	Family and social support		^30^^,^^31^^,^^32^
	Socio-familial barriers to appointment utilisation		^30^
Health and wellbeing factors	Medical factors and health conditions		^30^^,^^31^^,^^32^^,^^33^^,^^34^
	Mental, emotional, and psychological factors		^30^^,^^32^^,^^33^^,^^34^
Financial constraints and socioeconomic factors	Comprehensive financial challenges		^30^^,^^31^^,^^32^^,^^33^^,^^34^
Healthcare access and barriers	Transportation challenges and accessibility		^30^^,^^31^^,^^32^^,^^33^
	Geographical barriers in healthcare access	^30,32^	^31,32^

### Healthcare system determinants

This analytical theme captures how the provider–patient relationship and provider professionalism affect appointment utilisation. It highlights the impact of ineffective communication and negative interactions between healthcare providers and patients. Additionally, it encompasses various organisational factors within healthcare institutions, such as appointment scheduling and management. This analytical theme, has three descriptive themes, which are discussed below.

#### Provider–patient relationship and professionalism

Ineffective communication among healthcare providers, and between healthcare providers and patients, was identified as a key factor. This breakdown includes difficulties in rescheduling inadequate information about medical conditions, and negative encounters with staff.^30,32,34^ Patients reported that healthcare providers disregard their requests on appointment scheduling.^30^ Additional analysis identified verbal attacks by healthcare providers towards patients, providers' scheduling limitations owing to forgetfulness, and patients' dissatisfaction with providers' efficiency and professionalism.^30,32,34^ A patient stated: '*Sometimes I arrive late at the clinic because of lifts, and the nurses verbally attack me when that happens. So, eh … I become disappointed with the treatment after the effort made to arrive at the clinic’* (Participant 10, male, 44-years-old).

#### Healthcare organisation factors

Organisational challenges included appointment reminders being misplaced, received too early or too late. Appointment scheduling issues, such as extended waiting times, errors, cancellations, and inflexibility, were noted. Overloading patients with multiple appointments within a short period was identified as a cause of multiple non-utilised appointments. For example, a patient expressed their frustration, saying: *'... I mean I've had to come up here on the Tuesday because they wanted to check my weight, and then I think it was the Wednesday to have my eyes done, and I'm thinking, do I need to come up twice* [laughs]*'* (Patient 8, Region 1, Regular). Appointments timing issues, such as lengthy appointment duration at the clinic, waiting room delays, and consultation room delays, also contributed.^30–34^

#### Safety determinants of appointment utilisation

Additional analysis uncovered safety issues around attending appointments, including experiences of sexual assault and attacks reflecting broader societal issues. One participant reported: *'Seven men gang-raped me while I was walking back home from the clinic, stabbed me, and left me in the bush to die. A herdsman rescued me and called others from the village, and then they called an ambulance. I was taken to the hospital'* (Participant 11, female, 21-years-old).^30^

Healthcare system determinants mostly related to Levesque’s supply side (Table 3), although patient perspectives (demand-side) were also evident. Health system determinants align with Levesque’s framework dimensions: approachability, acceptability, availability and accommodation, appropriateness, and patients' ability to reach and engage. These dimensions illustrate how communication gaps and provider behaviour affect approachability, and individuals' willingness to engage. Organisational factors impact patients' needs, availability and accessibility, including challenges with reminders and appointments. This can lead to patient dissatisfaction and a lack of trust in the system’s ability to provide effective services, which could be perceived as inappropriate owing to general service provision inadequacies.

Moreover, the analysis highlights the challenges patients encountered on the way to appointments, highlighting safety concerns and the potentially dangerous process of accessing services. It also emphasised how patients' interactions with healthcare providers can influence their capacity and motivation to engage in care and utilise appointments effectively.

### Patient experience and decision making

This analytical theme includes the various personal factors that influence how patients utilise and engage with healthcare services. The analysis focused on patients' knowledge, awareness, and vulnerability, shedding light on the barriers they face and how these impact their healthcare experiences. The descriptive theme is personal factors and is discussed below.

#### Personal factors

Patients mentioned reasons for multiple missed appointments, including work commitments and forgetfulness. Varied patient knowledge and awareness about screening led to confusion about services, and appointment-making difficulties, contributing to multiple non-utilised appointments. Vulnerability owing to migrant documentation issues and access barriers were reported.^30–34^ For instance, one patient stated: *'*Pt*: Well, with being homeless for 8 weeks ... But they* [GP practice] *didn’t want to know. "Oh you’re not in our area." I’m in nobody’s area because we were in a bed and breakfast; they were my last doctors'* (Patient 10, Region 1, Non-regular). Here the patient was denied diabetic retinopathy screening through her general practice owing to her temporary housing status. Participants reported attending clinic appointments alone, adding to their difficulties.^30^ Furthermore, difficulties related to appointment booking methods and timing were encountered based on personal preference. Some individuals needed appointments on specific days and times based on personal preference, while others preferred pre-booked appointments and receiving their notifications digitally.^33,34^

Patient experience and decision making related to Levesque’s demand-side dimensions, particularly the ability to perceive, seek care, reach, and engage. This theme illustrates how personal factors influence patients' perceptions of care needs and their ability to navigate the healthcare system effectively. Patients' appointment preferences reflect individual needs and engagement levels in decision making within healthcare services, impacting appointment utilisation. Additionally, forgetfulness can hinder effective engagement, while patient autonomy and awareness influence decisions regarding attending appointments.

### Communication, support, and engagement

This analytical theme explores the social and familial factors that influence patients' ability to utilise healthcare services. It highlights how communication barriers, lack of support, and socio-familial dynamics can affect appointment attendance and engagement with healthcare providers. It includes three descriptive themes, which are discussed below.

#### Challenges with communication and language

Language challenges led to multiple missed appointments, with patients relying on family or close contacts for translation.^33^

#### Family and social support

Participants emphasised the lack of family and workplace support to attend appointments, with some dependent on them for access. This was especially evident when family members had competing priorities that made it difficult for individuals to attend appointments.^30–32^ Workplace stigma also affected attendance.^30^ For instance, a patient expressed: *'When I asked for time off to go to the clinic, my supervisor responded by saying "I hope you are not collecting AIDS pills". Sometimes he makes comments like "Are you also in the sinking ship of AIDS?"'* (Participant 28, female, 38-years-old).

#### Socio-familial barriers to appointment utilisation

Additional analysis identified barriers, including betrayal in relationships, social isolation, and stigma within families. This internalised bias may lead to multiple missed appointments.^30^ For instance, a participant expressed: *'My mother knows that I am HIV positive, but she* [mother] *prefers not to talk about it as it has brought shame to the family'* (Participant 5, male, 22-years-old).

The theme communication, support, and engagement is closely related to Levesque’s demand-side dimensions, particularly the ability to seek, reach, and engage with healthcare services. Patients' accounts illustrate how negative remarks can discourage individuals from seeking necessary medical care. Additionally, the absence of language support services and community outreach programmes to raise awareness creates barriers for patients in obtaining necessary understanding and assistance within the healthcare system. These limitations impact their ability to effectively engage with healthcare services.

### Health and wellbeing factors

This analytical theme explores how various health and wellbeing factors affect patients' attendance at healthcare appointments. Two descriptive themes were identified, which are discussed below.

#### Health conditions and medical factors

Patients and healthcare providers noted various physical health issues affecting appointment attendance. Concerns included memory impairment owing to post-traumatic stress disorder (PTSD), alongside multimorbidities, fatigue, and postoperative recovery.^30–34^ Multiple hospitalisations also emerged as a significant issue, highlighting the need for improved communication among healthcare providers regarding patients' health situations.^32^ For instance, in this study a mother said: '*My child is hospitalised a lot, so missed a lot of appointments, and then they kind of get forgotten*.'

Patients face challenges with side effects, such as vision impairment from eye drops, and practical issues, such as food deprivation during lengthy appointments, which is particularly problematic for patients with diabetes. Patient 5 (Region 3, Non-regular) recounted: *'Yes, the ﬁrst time I went to ... the local optician ... I was there for 5 hours, from 10 o’clock in the morning, and by the time I got out of the door it was 3 o’clock ... And by then I can remember I was so hungry and I thought, "well how does that help a diabetic person?".'* ^34^ The lengthy wait time of 5 hours, without consideration for their basic needs such as hunger, especially as a person with diabetes, suggests a lack of empathy and proper care.

#### Mental, emotional, and psychological factors

Patients' mental health significantly impacted their attendance at healthcare appointments. Challenges included past traumatic experiences, PTSD diagnoses, fear of travel and receiving bad health news, feelings of distress and humiliation disclosing financial difficulties, and discomfort with the physical proximity of healthcare staff.^30,32–34^

The health and wellbeing factors theme is associated with the demand-side dimensions of the ability to reach and engage within Levesque’s framework. These factors highlight the inadequacies in strategies to improve physical access and mental health support, resulting in low engagement with the healthcare system.

### Financial constraints and socioeconomic factors

This analytical theme explores the financial challenges experienced by patients, impacting their ability to attend appointments repeatedly. One descriptive theme emerged, which is comprehensive financial challenges.

#### Comprehensive financial challenges

Economic challenges presented a substantial barrier to maintaining regular appointment attendance, intensified by recent service policy changes reducing social welfare payments. Patients, especially those with a low socioeconomic status, grappled with job insecurity, which forced some to make difficult choices between attending appointments and generating income. Financial constraints also prevented patients from cancelling appointments in advance owing to a lack of funds for phone communication and transportation, resulting in multiple missed appointments, along with increased expenses related to gas and parking.^30–34^

This analytical theme is closely linked to Levesque’s demand-side dimensions, particularly the ability to pay, as found in all the included papers. These factors highlight inequitable access to healthcare services owing to socioeconomic disparities and insufficient financial support from organisations and the healthcare system.

### Healthcare access and barriers

This final analytical theme highlights obstacles to accessing healthcare services, including transportation challenges and the impact of geographical location on appointment utilisation. Patients residing far from healthcare facilities face logistical difficulties that hinder their ability to attend appointments. This theme encompasses two descriptive aspects, which are discussed below.

#### Transportation challenges and accessibility

Transportation challenges significantly hindered patients from attending healthcare appointments, including traffic issues, lack of personal cars, and the loss of transportation support from family owing to health conditions, particularly HIV. Public transport users faced additional challenges, such as wheelchair-accessibility difficulties, rigid schedules, and unreliable services, causing delays and missed appointments.^30–32,34^

#### Geographical barriers in healthcare access

Patients residing far from healthcare facilities faced logistical difficulties when trying to reach their appointments, making attendance more challenging.^30,32^ Additionally, inclement weather, posed a considerable challenge for patients to access healthcare appointments.^31,32^

The analytical theme of healthcare access and barriers is balanced between the supply side (healthcare system) and the demand side (patient experience). This theme relates to the dimensions of availability and accommodation, and the ability to reach within Levesque’s framework. It highlights the transportation challenges patients may encounter and the absence of alternative methods to ensure patients can access services. Furthermore, it underscores how logistical obstacles faced by patients living far from healthcare facilities impact their ability to attend appointments.

## Discussion

### Summary

The study explores factors influencing multiple non-utilised appointments across diverse global healthcare settings, drawing from five qualitative studies involving 151 participants, resulting in six emerged analytical themes. Four studies^30–33^ encompass all analytical themes. These insights offer a fresh perspective on a critical healthcare issue, revealing the interplay between supply (healthcare system) and demand (patients' experiences and/or perspectives).

On the supply side, building strong provider–patient relationships is key. Respectful and supportive interactions enhance patient engagement, while flexible appointment options improve access. Addressing workplace discrimination within healthcare settings appears to be crucial for creating a welcoming environment. Furthermore, tailoring services to individual needs, especially for those with specific health conditions, comes across as essential. Overcoming geographical barriers has an important role to play in ensuring universal access to healthcare services.

On the demand side, delivering safety and physical accessibility is paramount. Tailored interventions, such as flexible scheduling and effective reminders, improve healthcare utilisation. Addressing language barriers facilitates communication between patients and providers. Additionally, supporting vulnerable populations, including those facing documentation or accommodation issues, is essential. Providing mental health support and resources for emotional distress is likely to encourage healthcare engagement. Equitable access to healthcare services appears to be essential for improved health outcomes. Lastly, finding alternative transportation solutions and ensuring service access in adverse weather conditions are necessary for comprehensive healthcare delivery.

Understanding these complex factors, healthcare providers and policymakers can develop targeted interventions to address the specific needs of individuals facing multiple non-utilised appointments. This evidence review contributes to our understanding of the effectiveness and equity of healthcare services globally, underpinning the importance of using the concept of multiple non-utilised appointments. It highlights the sparsity of studies focused on missingness to date. Yet the study provides valuable insights and practical recommendations to enhance patient care and system efficiency, proving important not only for addressing immediate healthcare challenges, but also for making lasting contributions to the field.

### Strengths and limitations

While acknowledging the limited number of studies available for inclusion, despite there being no limitation on the year of publication, this comprehensive review spans a range of long-term conditions and healthcare settings globally. However, identifying relevant studies posed challenges owing to methodological inconsistencies, particularly in distinguishing between single and multiple instances of appointment non-utilisation. Although some quantitative studies offered insights into multiple non-utilised appointments, the overall data quality varied. One notable challenge encountered during the selection process was the reliance on researchers' experience to navigate the use of multiple terms, compounded by the absence of specific appointment number specification in the keywords tagged in papers.

### Comparison with existing literature

Our review identifies factors aligned with findings from research about single missed appointments. It highlights the crucial role of healthcare providers and systems in multiple non-utilised appointments including issues such as poor communication, negative provider interactions, and inconvenient schedules.^35–39^ Personal barriers, such as knowledge gaps, competing commitments, and forgetfulness, also contribute significantly to appointment challenges.^36,38–40^

Health-related factors, such as depression and other mental health diagnoses, align with evidence from previous studies.^40–44^ Financial and socioeconomic factors, including lower socioeconomic status and lack of insurance coverage, contribute to no-shows.^37,38,41–44^ Transportation challenges also emerge as a significant theme, consistent with prior reviews.^36,39^ Additionally, our review highlights geographical barriers in healthcare access, including clinic locations and weather conditions. While distance to the clinic can predict no-show behaviour in some settings.^39,41,43^

However, importantly our findings diverge from studies about single non-utilised appointments. We identify challenges in securing appointment times that suit personal needs, contrasting with findings suggesting scheduling may not be significant.^43^ Additionally, our review emphasises the vital impact of language barriers, perceived stigma, and lack of social support, consistent with prior literature on single missed appointments.^39,40^

In our review, health-related factors significantly influenced multiple non-utilised appointments. Patients often face challenges such as physical and mental health issues, and practical barriers such as long wait times and treatment side effects. This lines up with what previous research has shown about the connection between single missed appointments and these factors.^4,40,42^ However, while we previously mentioned distance as a reason for missed appointments, other studies found no association between clinic distance and attendance.^38^

### Implications for research

These findings will guide future research, particularly a qualitative study in a Gulf state country focusing on patients' and healthcare providers' perspectives regarding multiple non-utilised appointments. This research highlights the challenges patients face in accessing healthcare across different countries, underscoring the need for patient support in healthcare services. It is crucial to avoid stigmatising patients for not turning up for their appointments, as these arise for a range of complex reasons.

Understanding the complexity of multiple non-utilised appointments has significant implications for healthcare research and practice. It can provide a strong foundation for shaping policies and practices to benefit patients, providers, and the healthcare system. Insights into this phenomenon facilitate the development of targeted interventions to improve patient engagement, reduce disparities, streamline processes, and align systems with patient and provider preferences. These initiatives improve healthcare delivery effectiveness and efficiency, enhance patient experiences and outcomes, and guide evidence-based decision making to advance healthcare services.

## Trial registration number

PROSPERO CRD42023429465.

## Data

Materials and data used for the conduct of this research are available from the study authors on request.

## Reflexivity

The lead researcher, a female PhD student from Saudi Arabia, fluent in English and Arabic, has a background in medical administration training in Saudi Arabia and the USA. This review was supervised by two UK researchers: a Professor of General Practice and Inclusion Health, and a Professor of Behavioural Science and Health. The analysis incorporates both Eastern and Western perspectives, highlighting factors contributing to multiple non-utilizedutilised appointments. The team advocates for addressing these issues globally, as they are crucial in tackling health inequalities within healthcare systems.
